# Microbiome in Chronic Kidney Disease

**DOI:** 10.3390/life12101513

**Published:** 2022-09-28

**Authors:** Theodoros Tourountzis, Georgios Lioulios, Asimina Fylaktou, Eleni Moysidou, Aikaterini Papagianni, Maria Stangou

**Affiliations:** 1 Department of Nephrology, Hippokration Hospital, School of Medicine, Aristotle University of Thessaloniki, 54642 Thessaloniki, Greece; 2 Department of Immunology, National Peripheral Histocompatibility Center, Hippokration Hospital, 54642 Thessaloniki, Greece

**Keywords:** microbiome, microbiota, chronic kidney disease, dialysis, transplantation

## Abstract

The gut microbiome is a complex collection of microorganisms with discrete characteristics and activities. Its important role is not restricted to food digestion and metabolism, but extends to the evolution, activation and function of the immune system. Several factors, such as mode of birth, diet, medication, ageing and chronic inflammation, can modify the intestinal microbiota. Chronic kidney disease (CKD) seems to have a direct and unique effect, as increased urea levels result in alteration of the gut microbiome, leading to overproduction of its metabolites. Therefore, potentially noxious microbial uremic toxins, which have predominantly renal clearance, including p-cresyl sulfate, indoxyl sulfate and N-oxide of trimethylamine [Trimethylamine-N-Oxide (TMAO)], accumulate in human’s body, and are responsible not only for the clinical implications of CKD, but also for the progression of renal failure itself. Certain changes in gut microbiome are observed in patients with end stage renal disease (ESRD), either when undergoing hemodialysis or after kidney transplantation. The purpose of this review is to summarize the changes of gut microbiome and the protein bound uremic toxins which are observed in CKD and in different kidney replacement strategies. In addition, we attempt to review the connection between microbiome, clinical implications and immune response in CKD.

## 1. Introduction

Chronic kidney disease (CKD) is a progressive disease, with high morbidity and mortality in adult population. Its incidence is increasing constantly, and approximately 10% of people are affected by some form of CKD, which is associated with almost 1.2 million deaths worldwide. Cardiovascular disease is the main cause of death, followed by infections and malignancies. Hemodialysis, nonetheless remains the most frequently applied method, substituting kidney function, even though renal transplantation is still the method of choice [1].

CKD increases the risk of cardiovascular diseases, infections, malignancies, and its direct effect on the integrity of immune system have been extensively studied, during the last few years. Among possible explanations, Kidney-Gut axis has been recently proposed, and seems to play a unique and important role. The increased serum urea levels cause alterations of the intestinal flora which stimulate the production of gut-derived toxins and alter the intestinal epithelial barrier. These changes may lead to an acceleration of kidney damage [2]. The renal failure causes water and nitrogen waste retention, leading to disturbances of motility, secretion and absorption in the digestive tract. These irregularities conduce to the development of gut dysbiosis, followed by overproduction of toxic bacterial metabolites, with their displacement to the blood and development of endotoxemia. As a result, chronic kidney “low-grade” inflammation and oxidative stress develop, with further progression of kidney function in the vicious cycle of the kidney-gut axis [3]. Changes in the composition of the intestinal microbiome (dysbiosis) are also associated with chronic diseases, such as chronic inflammation, atheromatosis, chronic kidney disease, obesity and type 2 diabetes mellitus [4]. In comparison with other reviews, in the present study, we attempt to distinguish between the various changes of gut microbiome and protein bound uremic toxins in CKD and other kidney replacement strategies, with data from the last years.

Chronic kidney disease, as defined by Kidney Disease: Improving Global Outcomes (KDIGO) in their 2012 Clinical Practice Guidelines for Evaluation and Management of Chronic Kidney Disease, includes abnormalities of kidney structure or function, present for more than 3 months followed by implications affecting human organisms. It is defined as a glomerural filtration rate (GFR) less than 60 mL/min/1.73 m^2^ in combination by one or more markers of kidney dysfunction [5] including albuminuria (albumin excretion rate >30 mg/24 h, albumin to creatinine ratio >30 mg/g), abnormalities in urine sediment, electrolytic disorders due to tubular dysfunction, histological and structural abnormalities and/or history of kidney transplantation [6]. CKD is classified in 5 stages based on the estimated GFR and 3 stages based on albuminuria [5,6]. 

Kidney replacement strategies [hemodialysis, peritoneal dialysis (PD) or transplantation] are generally considered at stage G5, depending on the comorbidity, age etc. Almost 89% of end stage renal disease (ESRD) patients worldwide receive hemodialysis, while peritoneal dialysis is applied in a limited proportion of them [7]. Hemodialysis method and its alternatives, hemofiltration and hemodiafiltration, are based on the diffusion and convection phenomena, and on the use of biocompatible synthetic membranes [8]. PD, either as continuous ambulatory PD (CAPD) or automated PD, is based on solute transport diffusion and osmotic ultrafiltration, using the peritoneal membrane [9]. Renal transplantation is the treatment of choice for ESRD, it restores renal function and has been associated with better survival and reduced morbidity. However, immunosuppressive treatment may influence several parameters, including microbiota, immune function and susceptibility to infections [10].

## 2. Human Gut Microbiome and Body Metabolism

There are a huge number of microorganisms in the human body. This vast and complex collection of bacteria, including archaea, protists, fungi, viruses, and eukaryotic organisms coexist with the host in a specific environment, and may be commensal, symbiotic, or pathogenic, and this is defined as Microbiota. Microbiota, or microflora, as formerly called, is found in all multicellular organisms [11]. The term microbiome is more generic, and includes microorganisms with their genes and also, the environment with its distinct physicochemical properties. There are other definitions, such as genetic, including a set of microbe genes in a host, or ecological definitions, referring to a set of microorganisms, and their genomes in a specific habitat. However, the former one is considered as the most comprehensive [12]. The microbiome is dynamic and transforms during humans life, under the influence of factors such as diet, environment, medical interventions and diseases [13]. The microbiota differs between people, and between different parts of the human body, in its vast majority is anaerobic and found mainly in the gut, especially the colon, which has a rich nutrient environment [14].

The gut microbiota is composed of more than 1500 species, in more than 50 phyla. The most dominant phyla are *Bacteroidetes* and *Firmicutes*, followed by *Proteobacteria*, *Fusobacteria*, *Tenericutes*, *Actinobacteria* and *Verrucomicrobia*. The healthy intestinal microbiome consists mainly by gram positive *Firmicutes*, gram negative *Bacteriodetes* and gram positive *Actinobacteria* [15]. All of the above are up to 90% of the total human microbial population. Each part of the gastrointestinal tract is colonized by specific species of microbiome. In healthy individuals, stomach is normally colonized by *Lactobacillus* and *Helicobacter*, duodenum by *Staphylococcus*, *Streptococcus* and *Lactococcus*, jejunum by *Enterococcus*, *Streptococcus* and *Lactobacillus*, ileum by *Enterobacteriaceae*, *Bacteroides*, *Clostridium* and segmented filamentous bacteria and the colon by *Firmicutes*, *Bacteroidetes*, *Actinobacteria*, *Proteobacteria*, *Clostridium*, *Lactobacillaceae*, *Prevotellaceae* and *Fusobacteria* [16]. 

Factors that can change the intestinal microbiota are host genetics, diet, age, mode of birth and antibiotic treatment [17]. Vegetarian diets are related with the dominance of *Firmicutes* and *Bacteroidetes*. A diet rich in protein and fats, is correlated with a plethora of bile-tolerant species (*Bacteroides*, *Bilophila*, *Alistipes*) and a reduction of *Firmicutes* [18]. *Firmicutes*, *Actinobacteria*, *Tenericutes* and *Euryarchareota* are more heritable, while most of the *Bacteroidetes* have very little heritability potential [19]. The initial colonizers of the intestine in infants are acquired through microbe contact deriving from the mother and the environment. There is an association between the infants’ microbiome and the route of delivery. Infants delivered vaginally, accommodate microbial communities, especially *Lactobacillus* spp. and *Bifidobacterium* spp., which are similar to the maternal vaginal canal. Infants delivered by cesarean section harbor microbial communities of skin microbes such as *Staphylococcus* [13]. As the time passes, the microbiota changes gradually, and the anaerobic population, mainly the *Bifidobacterial* population and *Bacteroides* are substituted by the *Enterobacterial* population. Moreover, an amylolytic activity and short-chain fatty acids (SCFA) production are also reduced [18]. Antibiotic treatment also affects gut microbiota, for example, clarithromycin treatment, frequently used against *Helicobacterpylori* causes a reduction in the number of *actinobacteria*, and ciprofloxacin reduces *Ruminococcus* [20]. Vancomycin decreases *Bacteroidetes*, *Fuminococcus*, *Faecalibacterium* and increases *Proteobacteria* species [21].

The utility of gut microbiome extends in many different aspects in human’s life, not all of them been fully investigated. Apart from the participation in plant derived polysaccharides and dietary oxalates, it seems to contribute in vitamins synthesis and immune system development [2]. The gut microbiome participates in food digestion mainly through two catabolic pathways, saccharolytic and proteolytic [22]. In the saccharolytic pathway, the gut microbiota breaks down sugars, contributing to the production of SCFA. In the proteolytic pathway, proteins are fermented or fragmented (protein fermentation), resulting in the production of both SCFA and other metabolites, such as ammonia, various amines, thiols, phenols and indoles. Some of these metabolites have predominantly renal clearance. Therefore, in the presence of renal dysfunction, they accumulate in human’s body, their serum levels are increased, and, as they are potentially toxic, they are called or considered as microbial uremic toxins [14,23]. The European Uremic Toxin Work Group sorts uremic toxins according to their physicochemical properties into three categories: free water-soluble low-molecular-weight solutes (<500 Dalton), protein bound solutes and middle sized molecules (≥500 Dalton) [24]. The main uremic toxins, including p-cresyl sulfate, indoxyl sulfate and N-oxide of trimethylamine [Trimethylamine-N-Oxide (TMAO)] [25,26] are highly, more than 80%, bound to proteins. Intestinal microflora facilitate the breakdown of tyrosine/phenylalanine and tryptophan into p-cresyl and indole respectively. Subsequently, after their absorption, oxidation and conjugation with sulfate follows, resulting in p-cresyl sulfate and indoxyl sulfate respectively. In circulation, they bind to albumin, so they cannot be removed through glomerular filtration. The only route of elimination is through the tubular transport system, which is lost as CKD progresses [27]. Gut microflora converts various nutrients (choline, betaine, L-carnitine and others from meat, fish, eggs, dairy products) into trimethylamine (TMA). Subsequently, TMA enters the circulation and is oxidized to TMAO by hepatic flavin-containing monooxygenase [28].

Therapeutic manipulation of the gut microbiome is achieved through modification of the diet, administration of prebiotic and/or probiotic supplementation, antibiotics (metronidazole, tylosin, rifaximin), fecal microbiome transplantation [13], drugs that absorb bacterial derived toxins in the digestive lumen and medications used before for other indications (acarbose, meclofenamate, lubiprostone) [3]. In CKD patients, these treatments are used to alterate the gut microbiota [23]. In a recent review, an adequate and appropriate dietary fiber intake to rehabilitate beneficial intestinal microbiota composition that would reduce the risks and implications associated with CKD, such as diabetes, cardiovascular diseases, obesity and cancer, is recommended [29].

## 3. Microbiome in Chronic Kidney Disease

### 3.1. Microbiome in CKD (at Pre-Dialysis Stages)

As CKD progresses, the urea concentration in blood gradually increases. As renal function declines, the gastrointestinal tract becomes the main route for urea excretion [30]. In advanced CKD, is observed impaired intestinal barrier function and chronicinflammation throughout the digestive tract. Urea diffuses from theblood into the gut lumen, stimulating the overproduction of urease containing bacteria, as an attempt to facilitate its catabolism [31]. Luminal urea is converted to ammonia, by microbial urease, and subsequently to ammonium hydroxide, that causes disruption of the epithelial barrier and increase of gut permeability, thus allowing translocation of gut uremic toxins, endotoxins, antigens and gut microorganisms or other microbial products into circulation. This phenomenon called “atopobiosis” (i.e., the microbes that appear in places other than their normal location), is associated with inflammatory diseases and is recognized as a route of endogenous infections [32]. Additional possible mechanisms contributing to the alteration of gut microbiome in CKD are associated either with the primary disease or comorbidities, such as presence of diabetes mellitus, medications, including phosphate binders, or eating habits, especially reduced fiber intake [2]. In several studies, the accumulation of uremic toxins is associated with the pathophysiology of CKD complications [33], such as vascular calcification, atherosclerosis [34], anemia [35], insulin resistance [36] and bone disorders. Whether these toxins are involved in immune system dysfunction in CKD is not well established [37]. Moreover, gut dysbiosis is starting to be recognized as a non-traditional factor for cardiovascular risk in CKD [38]. The association between kidney-gut axis and gut microbiome is bidirectional. The changes in gut environment cause gut dysbiosis [39]. The interaction between them is a bidirectional relationship, as CKD leads to a shift of healthy intestinal microbiome to a condition of imbalance between healthy and pathogenic bacteria called gut dysbiosis. The gut dysbiosis disrupts the epithelial integrity of intestine, intensifies inflammatory and immunological processes due to endotoxemia, gut derived uremic toxins, and acidosis which leads to progression and complications of CKD [40]. The specific CKD diet usually recommended is low in sodium, potassium and phosphate intake, resulting in reduced absorption of substantial nutrients, such as dietary fibers. Dietary fibers produce SCFA, which protect against damage of the intestine [41]. As renal function deteriorates, causes retention of uremic toxins. These toxins contains urea accumulate not only in the intestine but in the blood and promote the colonization of microbes that can use urea as an energy source [42].

In patients with CKD, the gut microbiota has already undergone substantial changes, regarding mainly the balance of the intestinal microbiome (Table 1). Normal colonic microbiota, including *Lactobacillaceae* and *Prevotellaceae* is significantly reduced, while *Enterobacteria* and *Enterococci*, normally present, though in small numbers, are increased up to 100 times in CKD patients [43]. Additionally, an overgrowth of *Enterobacteriaceae*, *Lachnospiraceae* and *Ruminococcaceae* observed together with a reduction of some *Bacteroidaceae*, *Prevotellaceae* and particularly *Bifidobacterium* and *Lactobacillus* species, have been described in CKD [38].

As kidney function declines, indoxyl sulfate is increasing in the blood, due to its limited kidney excretion, leading to further deterioration of CKD [44]. In proximal tubule cells, indoxyl sulfate activates nuclear factor kappa-light-chain-enhancer of activated B cells (NF-κB). As a result, cellular proliferation is suppressed; senescence is induced and accelerated through induction of p53. Furthermore, fibrosis is stimulated by expression of transforming growth factor beta 1 (TGF-β1) and plasminogen activator inhibitor-1 (PAI-1) [45]. Quorum sensing is a bacterial regulatory mechanism that perceives and stimulates synchronized behaviors. It depends on bacterial or other cells population density. It operates through the secreted molecular compounds called quorum sensing signals [46]. These signals can be created either by pathobionts or by autochthonous microbiota. Those produced by gram negativebacteria, such as Pseudomonas aeruginosa have negative immune related actions such as activation of mitogen activated protein kinase pathways. These induce NF-kB signaling and chemotaxis. Subsequently, they increase inflammatory genes expression [47]. Moreover, indoxyl sulfate induces an epithelial mesenchymal transition of tubular epithelial cells through activation of the renin angiotensin system, which contributes to renal fibrosis [48]. In addition, indoxyl sulfate induces Klotho depletion. Klotho is an anti-aging gene with renal protective attributes [49]. The mechanism associated with progression of CKD from p-cresyl sulfate is similar to indoxyl sulfate [45]. Furthermore, p-cresyl sulfate inhibits efflux transporters multidrug resistance protein 4 (MRP4) and breast cancer resistance protein (BCRP) in proximal tubular cells, causing an intracellular accumulation of toxins, including p-cresyl sulfate [50]. TMAO may also contribute to the progression of CKD by promoting renal fibrosis, specifically tubulointerstitial fibrosis, and increasing expression of pro-fibrotic genes and kidney injury markers [45].

**Table 1:** Gut microbiome in CKD and kidney replacement strategies.

	Increase/Growth	Decrease/Reduction	Authors, Year, Type of Study
Pre-dialysis CKD	*Enterobacteriae* [38,43] *Enterococci* [43] *Lachnospiraceae* [38] *Ruminococcaceae* [38]	*Lactobacillaceae* [38,43] *Prevotellaceae* [38,43] *Bacteroidaceae* [38] *Bifidobacterium* species [38]	Sampaio-Maia et al. [38], 2016, review Vaziri et al. [43], 2013, cohort study (*n* = 24)
Hemodialysis	*Proteobacteria*(mainly *Gammaproteobacteria* [43,51]) *Actinobacteria* [43,51] *Firmicutes* [43,51,52] (mainly *Clostridium*, *Enterococcus*)	*Lactobacillaceae* [53] *Prevotellaceae* [53]	Vaziri et al. [43], 2013, cohort study (*n* = 36) Chen at al. [51], 2019, review Shi et al. [52], 2014, cohort study (*n* = 52) Wong et al. [53], 2014, cohort study (*n* = 24)
Peritoneal dialysis	*Proteobacteria* [54] *(Pseudomonas* *aeruginosa)* [55]	*Actinobacteria* [56] *Firmicutes* [56] *Lactobacillaceae* [55] *Bifidobacterium* species [55]	Crespo-Salgado et al. [54], 2016, cross-sectional study (*n* = 39) Wang et al. [55], 2012, cohort study (*n* = 29) Simões-Silva et al. [56], 2020, cross-sectional study (*n* = 20)
Kidney transplantation	*Proteobacteria* [57,58]	*Actinobacteria* [58]	Lee at al. [57], 2014, pilot study (*n* = 26) Swarte et al. [58], 2020, cohort study (*n* = 139)

### 3.2. Microbiome in Hemodialysis

*Proteobacteria* (mainly *Gammaproteobacteria*), *Actinobacteria* and *Firmicutes* are increased in hemodialysis patients [43,51], although some investigators showed that *Proteobacteria* are significantly reduced in hemodialysis compared to peritoneal dialysis patients [32] (Table 1). Even though, pediatric hemodialysis patients have significantly increased levels of indoxyl sulfate and p-cresyl sulfate, no differences were observed in this taxa due to the production of these uremic toxins, namely *Bifidobacteriaceae*, *Clostridiaceae*, *Enterobacteriaceae* and *Lactobacillaceae* [54].

Apart from gut, other microbiomes have also been studied in hemodialysis population, which revealed an increased proportion of microbial colonization among hemodialysis patients. In the blood samples of almost 21% of hemodialysis patients (either from peripheral vein or arteriovenous fistula or central venous catheter), bacterial DNA has been found the species included *Escherichiacoli*, *Staphylococcusaureus*, *Pseudomonasaeruginosa*, *Staphylococcusepidermidis*, *Enterococcusfaecalis*, *Proteusmirabilis* and *Staphylococcushaemolyticus* [59]. Additionally, in other studies in ESRD (pre-dialysis and hemodialysis) the blood colonization was *Firmicutes*, *Bacteroidetes* and *Proteobacteria*, concerning the bacteria phylum. At genus level, the dominant bacteria were *Escherichia*, *Shigella*, *Prevotella*, *Faecalibacterium*, *Bacteroides* and *Ruminococcus* [52].

Dominant microbiomes’ families in ESRD (i.e., *Clostridiaceae* and *Enterobacteriaceae*) possess urease, uricase, indole and p-cresyl forming enzymes. As a result, more uremic toxins derive, which may contribute to uremic toxicity and inflammation. On the other hand, a reduction is found for *Lactobacillaceae* and *Prevotellaceae*, which produce butyrate forming enzymes, that can affect the production of butyrate, and has beneficial effect on the intestine [53]. A study showed that the SCFA (propionate, acetate, butyrate), selectively enlarge the pool of the regulatory T cells in the large intestine. The expansion of these cells by the SCFA, assists to downregulate inflammation by suppressing the operation of inflammatory cells [60]. The excessive ultrafiltration volume and/or intradialytic hypotension can cause episodes of transient intestinal ischemia. As a result, these may impair the function and permeability of intestinal barrier in patients on dialysis [61].

The highest levels of p-cresyl sulfate and indoxyl sulfate in blood are observed in hemodialysis patients. Clearance of indoxyl sulfate and p-cresyl sulfate by hemodialysis is limited, as both molecules display very high protein binding ratios (more than 95%), which cannot be successfully removed by hemodialysis membranes, leading to the accumulation of uremic toxins [62]. Hemodialysis reduction rates of these uremic toxins, even by using high-flux membranes, are estimated around 35%. Removal of these can be meliorated (to some extent) by raising the diffusion of the free, unbound molecules with super-flux membrane, increasing the dialyzer mass transfer area coefficient and dialysate flow, haemodiafiltration, daily sessions and addition of a sorbent to dialysate [63]. TMAO accumulates in hemodialysis. Peak levels are almost 40-fold than in normal population. This is principally due to two factors. First, TMAOs’ clearance in normal kidney is almost four-fold with respect to urea, while its clearance by dialysis is less than that of urea. The ratio of dialytic to normal clearance is much lower for TMAO than for urea. Secondly, the TMAO has lower volume of distribution than urea. So the inefficiency that results from the intermittency of classic dialysis treatment is larger than for urea [64].

### 3.3. Microbiome in Peritoneal Dialysis

As peritoneal dialysis, in most countries, is far less frequently used as a kidney replacement therapy, studies in this population are lacking. In patients undergoing peritoneal dialysis, there is a decrease in *Actinobacteria* and *Firmicutes* [56]. Peritoneal dialysis patients are unlikely to have *Bifidobacteriumcatenulatum*, *Bifidobacteriumlongum*, *Bifidobacteriumbifidum*, *Lactobacillusplantarum*, *Lactobacillusparacasei* and *Klebsiellapneumonia* [55] (Table 1). Both *Lactobacillus* and *Bifidobacterium* participate in the regulation of gut microbial homeostasis and possibly reduce the constipation rate. So, these reduced populations can be associated to some adverse effects [32]. In a study from Wang et al., there was an increased prevalence of *Pseudomonasaeruginosa* in the fecal samples of patients undergoing peritoneal dialysis [55]. *Pseudomonas* is a possible agent for peritonitis, and responsible for almost 40% of catheter removal related to infection [32].

The renal clearance of indoxyl and p-cresyl sulfate are positively correlated with the renal clearance of urea nitrogen and creatinine. So, there is a significant role of residual renal function in the removal of these uremic toxins. Additionally, these solutes could not be removed efficiently, even after increasing the PD dose or altering the state of the peritoneal membrane. A study of 57 patients with end stage renal disease on peritoneal dialysis showed that sevelamer could be a helpful approach to reduce p-cresyl circulating levels in this population. This may also affect cardiovascular risk due to its anti-inflammatory effect [65]. Another study revealed that indoxyl sulfate serum concentration is considerably lower in patients on CAPD than those on low flux hemodialysis, a finding that can be attributed to residual renal function, as this was an independent parameter with inverse correlation with indoxyl sulfate serum concentration [66].

### 3.4. Microbiome in Kidney Transplantation

Transplantation in general, induces an unbalanced dysbiotic gut microbiome, characterized by a loss of microbial diversity and an increase in the *Proteobacteria* and reduction in *Actinobacteria* phylum [57,58,67] (Table 1). These microbial changes seem to persist up to 6 years after renal transplantation [58]. *Proteobacteria* (which are plenty in dysbiosis), can act as pathobionts. They normally show no pathogenic behavior in a healthy gut; however, under certain circumstances, they may become colitogenic pathogens and trigger local and systemic inflammation. These conditions are characterized by an impermanent increase in oxygen levels, which can favor the increase of facultative anaerobes, such as *Proteobacteria* [68]. Certain *Proteobacteria* induce a pro-inflammatory state linked with allograft rejection [67], while others report a reduction in the *Bacteroidetes* phylum during the rejection episode [57]. The main factors that seem to determine gut microbiome in transplant patients are immunosuppression and renal graft function. Mycophenolate mofetil and tacrolimus can reduce gut microbial diversity, in favor of pathobionts that can induce gastrointestinal toxicity [58,67].

In two studies in kidney transplanted patients, serum levels of indoxyl and p-cresyl sulfate decreased considerably following renal transplantation. The post-transplantation levels of these toxins were lower than in a non-transplant person with identical GFR. This is explained by the administration of immunosuppressants, antibiotics or other drugs (that can alter gut microbiome) or the transplantation procedure itself (that may change the composition of the colonic microbiota) [69,70]. Elevated TMAO levels are strongly associated with the degree of renal function in CKD and are normalized after kidney transplantation and remain low for at least 2 years [26].

## 4. Microbiome and Clinical Implications (Table 2)

Pre-inflammatory and pre-oxidant effects of indoxyl sulfate may be implicated in increased frequency of atheromatosis and vascular calcification, leading to cardiovascular disease and increased morbidity and mortality among ESRD patients [71]. P-cresyl sulfate induces endothelial damage, retards endothelial repair and increases senescence of mature endothelial cells, thereby increasing cardiovascular and total mortality [72]. It also activates the renin, angiotensin, aldosterone system and causes disorders in the epithelium, which contribute to fibrosis and the progression of kidney damage [48]. Blood levels of p-cresyl sulfate and indoxyl sulfate are associated with worsening renal function and all-cause mortality in patients with various stages of CKD [73]. In a study of 333 hemodialysis patients, increased levels of indoxyl sulfate and p-cresyl sulfate are associated with an increased risk of acute coronary syndrome. The proteins were related with cardiovascular related proteins, which are involved in endothelial barrier function, complement system, cell adhesion, phosphate homeostasis and inflammation. Furthermore, free form of indoxyl sulfate had a positive correlation with fibroblast growth factor 23 (FGF23). High serum FGF23 levels are positively linked with all-cause mortality and cardiovascular events in hemodialysis population [74]. In a recent study, it was observed that hemodiafiltration was not superior to classical hemodialysis in the removal of specific uremic toxins. In the same study, no statistically significant relationship was observed between the accumulation of these toxins with total mortality and cardiovascular events, over a 6-month follow-up period [75]. 

TMAO levels are associated with increased systemic inflammation, cardiovascular events and mortality not only in patients undergoing hemodialysis, but also in the presence of CKD stage 3–5 [26]. In PD patients, results are controversial; however, in most studies, TMAO serum levels have been correlated with cardiovascular mortality and peritoneal infection. No obvious connection between TMAO and all-cause mortality observed [76,77].

**Table 2:** Association between gut microbiome and clinical implications in CKD.

	Clinical Implications	Authors, Year, Type of Study
CKD	atheromatosis [34,71] vascular calcification [34] anemia [35] bone disorders [78] progression of CKD [45] systemic inflammation [26] cardiovascular risk factor [38]	Opdebeeck et al. [34], 2019, cohort study (*n* = 42) Hung et al. [71], 2017, review Chiang et al. [35], 2011, laboratoty cohort study Lin et al. [78], 2014, cohort study (*n* = 80) Lim et al., 2021, review Missailidis et al. [26], 2016, prospective cohort study (*n* = 179) Sampaio-Maia et al. [38], 2016, review
ESRD	systemic inflammation [26] cardiovascular mortality [72] all cause mortality [72]	Missailidis et al. [26], 2016, prospective cohort study (*n* = 179) Graboski et al. [72], 2020, review
Hemodialysis	acute coronary syndrome [74] cardiovascular events [26,74] systemic inflammation [26] all cause mortality [26,74]	Wu et al. [74], 2021, cross-sectional study (*n* = 333) Missailidis et al. [26], 2016, prospective cohort study (*n* = 179)
Peritoneal dialysis	peritoneal infection [76,77] cardiovascular mortality [76]	Chang et al. [76], 2021, prospective cohort study (*n* = 513) Zhang et al. [77], 2022, cohort study
Kidney transplantation	allograft rejection (possible) [67]	Baghai Arassi et al. [67], 2020, review

## 5. Microbiome and Immune Reactions

Concerning the immune system development, the *Bacteroidesfragilis* polysaccharide A has the ability to cultivate T helper (Th) cell ratio (Th1/Th2). Dendritic cells and lymphoid tissues associated with the gut in the gastrointestinal tract components of *Bacteroidesfragilis*, migrate to lymphoid organs, and promotes Th1 lineage differentiation [79].

P-cresyl sulfate is associated to an immune deficiency condition of CKD, mostly correlated to the adaptative immune response. Indoxyl sulfate is related to the activation of innate and adaptative immune system, possibly responsible of the CKD associated inflammation [80]. In patients with CKD, a correlation is observed between p-cresyl sulfate and a reduction of B lymphocyte population, while there is not enough evidence on the effect of uremic toxins on naive or differentiated T cells [37]. P-cresyl sulfate and indoxyl sulfate cause an increased adhesion of neutrophils to endothelial cells and their extravasation [81]. Furthermore, p-cresyl sulfate reduces phagocytotic activities of monocytes, macrophages and dendritic cells, while in the latter it also reduces antigen presentation [82]. P-cresyl sulfate exerts a negative effect in Th1 lymphocytes, leading to reduced production of interferon-γ (IFN-γ) [83]. Indoxyl sulfate causes pro-inflammatory effects, endothelial dysfunction and bone disorders [72]. Indoxyl sulfate-induced pro-inflammatory macrophage activation is mediated by its uptake through transporters, including organic anion transporting polypeptides 2B1 (OATP2B1), encoded by the solute carrier organic anion transporter 2B1 (SLCO2B1) gene [84]. Indoxyl sulfate increases levels of tumor necrosis factor α (TNF-α) and interleukin 6 (IL-6) and causes an exacerbation of the inflammatory condition through the promotion of oxidative stress [85].

TMAO accumulates in the heart, kidney, and other tissues, participating in various biological processes, such as activation of platelet aggregation, increase in foam cell formation, activation of inflammatory responses, and reduction in reverse cholesterol transport [28]. Accumulation of uremic toxins favors the development of immunological disorders in ESRD, mainly associated with T lymphocyte disorders [86]. Serum concentration of TMAO has been positively correlated with C-reactive protein levels and increased concentration of IL-6 and PAI-1, in peritoneal dialysis fluid, within PD patients. Furthermore, in addition to high glucose-induced TNF-α and chemokine (C-C motif) ligand 2 (CCL2) expression in endothelial cells, TMAO may trigger TNF-α-induced P-selectin production in mesothelial cells, and thus can directly induce peritoneal mesothelial cell necrosis, together with increased production of pre-inflammatory cytokines, including CCL2, TNF-α, IL-6, and IL-1 [77].

## 6. Conclusions

**Figure 1:**
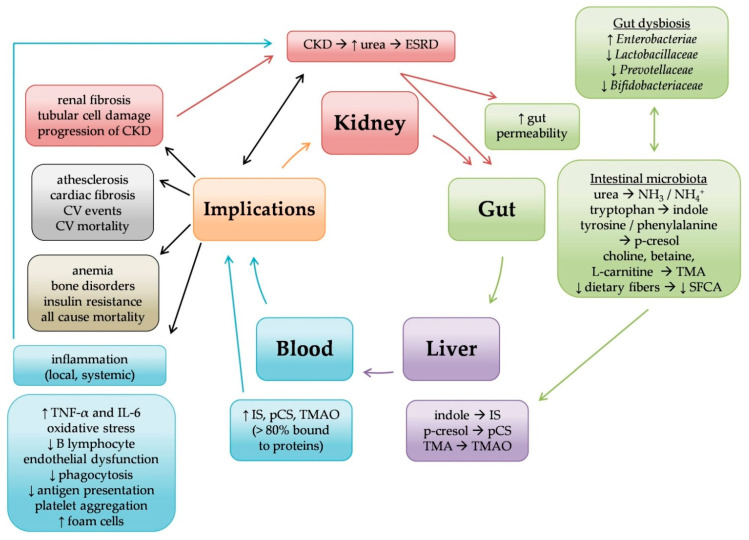
Relationship between kidney-gut axis, gut microbiome and implications in chronic kidney disease (CKD). As renal function declines, urea accumulates in the bloodstream and diffuses into the gut lumen. Urea is converted to ammonia and subsequently to ammonium hydroxide; this causes disruption of the epithelial barrier and increases the gut permeability (red arrows above inscription “kidney”). Certain bacteria (green double arrow) metabolize urea and other diet products, leading to overproduction of protein bound uremic toxins, such as indoxyl sulfate (IS), p-cresyl sulfate (pCS) and Trimethylamine-N-Oxide (TMAO) (green arrow). Moreover, the production of anti-inflammatory short-chain fatty acids (SCFA) is reduced. The accumulation of uremic toxins (blue arrow) is associated with renal, cardiovascular (CV) and other implications (black arrows). Renal fibrosis (left red arrow) and local inflammation (left blue arrow) promote progression of CKD. So, a vicious cycle in kidney-gut axis is formed (curved arrows). Each color represents different organ or category: red, kidney; green, gut; purple, liver; blue, blood; grey, cardiovascular implications; brown, other implications. ESRD, end stage renal disease; TNF-α, tumor necrosis factor α; IL-6, interleukin 6.

The intestinal microbiome has a significant role in human functions and in a variety of diseases. It is tangled up with renal failure in a vicious circle (Figure 1). Different microbiomes’ families, which possess uremic toxins forming enzymes, in CKD and ESRD are either increased or decreased. As a result, more p-cresyl sulfate, indoxyl sulfate and TMAO are derived, which could contribute to uremic toxicity and inflammation. As described, gut microbiome has a direct correlation with CKD progression and its implications, such as cardiovascular events, systemic inflammation and mortality, making it a potential therapeutic objective. Future perspectives include correlation with other diseases in CKD, such as cancer or certain infections. Further studies are necessary, aiming to find better ways to remove protein bound uremic toxins or other therapeutic manipulations, which may improve morbidity and mortality of CKD patients.

## Data Availability

Not applicable. No new data were created or analyzed in this study.
